# MMSE-Based Dementia Prediction: Deep vs. Traditional Models

**DOI:** 10.3390/life15101544

**Published:** 2025-10-01

**Authors:** Yuyeon Jung, Yeji Park, Jaehyun Jo, Jinhyoung Jeong

**Affiliations:** 1 Department of Dental Hygiene, College of Medical Science, Konyang University, Daejeon 35365, Republic of Korea; yuyeon@konyang.ac.kr; 2 Department of Medical Engineering, College of Medical Convergence, Catholic Kwandong University, Gangneung-si 25601, Republic of Korea; yj2023@cku.ac.kr (Y.P.); jh_507@cku.ac.kr (J.J.); 3 Department of Biomedical Management, College of Medical Convergence, Catholic Kwandong University, Gangneung-si 25601, Republic of Korea

**Keywords:** dementia prediction, deep learning, MMSE, explainable AI, cognitive assessment

## Abstract

Early and accurate diagnosis of dementia is essential to improving patient outcomes and reducing societal burden. The Mini-Mental State Examination (MMSE) is widely used to assess cognitive function, yet traditional statistical and machine learning approaches often face limitations in capturing nonlinear interactions and subtle decline patterns. This study developed a novel deep learning-based dementia prediction model using MMSE data collected from domestic clinical settings and compared its performance with traditional machine learning models. A notable strength of this work lies in its use of item-level MMSE features combined with explainable AI (SHAP analysis), enabling both high predictive accuracy and clinical interpretability—an advancement over prior approaches that primarily relied on total scores or linear modeling. Data from 164 participants, classified into cognitively normal, mild cognitive impairment (MCI), and dementia groups, were analyzed. Individual MMSE items and total scores were used as input features, and the dataset was divided into training and validation sets (8:2 split). A fully connected neural network with regularization techniques was constructed and evaluated alongside Random Forest and support vector machine (SVM) classifiers. Model performance was assessed using accuracy, F1-score, confusion matrices, and receiver operating characteristic (ROC) curves. The deep learning model achieved the highest performance (accuracy 0.90, F1-score 0.90), surpassing Random Forest (0.86) and SVM (0.82). SHAP analysis identified Q11 (immediate memory), Q12 (calculation), and Q17 (drawing shapes) as the most influential variables, aligning with clinical diagnostic practices. These findings suggest that deep learning not only enhances predictive accuracy but also offers interpretable insights aligned with clinical reasoning, underscoring its potential utility as a reliable tool for early dementia diagnosis. However, the study is limited by the use of data from a single clinical site with a relatively small sample size, which may restrict generalizability. Future research should validate the model using larger, multi-institutional, and multimodal datasets to strengthen clinical applicability and robustness.

## 1. Introduction

According to the World Health Organization (WHO), as of 2021, approximately 57 million people worldwide were living with dementia, with more than 60% living in low- and middle-income countries. Furthermore, nearly 10 million new cases of dementia are diagnosed each year. Dementia is emerging as a major public health issue as the global population ages. As of 2023, there are approximately 55 million people worldwide suffering from dementia, and this number is expected to reach 152 million by 2050 [1]. Dementia not only significantly reduces the quality of life for patients but also imposes substantial economic and psychological burdens on families and society as a whole, underscoring the growing importance of early diagnosis and prediction.

In clinical settings, standardized cognitive assessment tools such as the Mini-Mental State Examination (MMSE) are widely used for early diagnosis of dementia [2]. While the MMSE is a simple and reliable test, it has limitations in capturing the complex patterns of cognitive decline observed in patients with diverse causes and backgrounds in real clinical settings. Previous studies have primarily relied on statistical analyses based on MMSE total scores or individual item scores, combined with traditional machine learning techniques such as Random Forest and Support Vector Machine (SVM). However, these approaches have limitations in adequately reflecting nonlinear interactions between variables, high-dimensional features, and subtle cognitive change patterns [3].

Recently, deep learning-based artificial intelligence technology has been gaining attention in the medical field for its ability to effectively learn complex patterns and nonlinear relationships from large-scale clinical data [4]. Deep learning has the advantage of being able to detect early signs of dementia that are difficult to capture with existing statistical or machine learning techniques by learning subtle interactions and potential predictive signals between various cognitive function assessment items. In fact, recent studies have reported that deep learning models demonstrate higher performance than existing methods in dementia prediction and diagnosis [5].

However, there are few studies that directly compare the performance of high-dimensional deep learning models with traditional machine learning models using MMSE raw data collected from actual clinical environments in Korea, and simultaneously present clinical interpretability by applying Explainable AI techniques [6,7,8,9].

Therefore, this study aims to develop a high-dimensional deep learning-based dementia prediction model using MMSE data and evaluate the validity of applying deep learning by comparing its performance with traditional machine learning (Random Forest, SVM) models. Additionally, by applying SHAP (Shapley Additive exPlanations)-based Explainable AI techniques, we aim to deeply analyze the model’s predictive rationale and clinical interpretability, thereby establishing new research and practical standards in the field of early dementia diagnosis.

## 2. Methods

### 2.1. Dataset and Data Collection

This study utilized MMSE (Mini-Mental State Examination) data provided by the AI Hub, a publicly available national AI dataset platform supported by the Korean government. The dataset was constructed by domestic medical institutions under prior ethical approval and anonymized before release. Since the research team only accessed and analyzed anonymized secondary data, no additional ethical approval was required for this study, consistent with international guidelines on secondary data use (e.g., Declaration of Helsinki and local IRB exemption policies). Approximately 164 participants, ranging from healthy individuals aged 55 and older to those with mild cognitive impairment and dementia, were recruited to collect a cognitive function dataset aimed at assessing the degree of cognitive decline and identifying which specific cognitive functions were most severely impaired. The data consists of 44 items, including demographic information of the study subjects, diagnosis, number of tests, individual MMSE item scores (Q01–Q19), and total score (TOTAL).

The target variable, diagnosis name (DIAG_NM), was classified into three groups: normal, mild cognitive impairment (MCI), and dementia. During the analysis process, this variable was converted into a numerical label using LabelEncoder.

During preprocessing, we identified 14 rows (≈4.7% of the raw dataset) containing missing values, which were removed prior to analysis. While imputation methods such as mean substitution and k-nearest neighbor imputation were considered, we excluded incomplete cases to prevent introducing artificial bias into small-scale data.

### 2.2. Model Development and Training

#### 2.2.1. High-Dimensional Deep Learning Model

**Figure 1:**
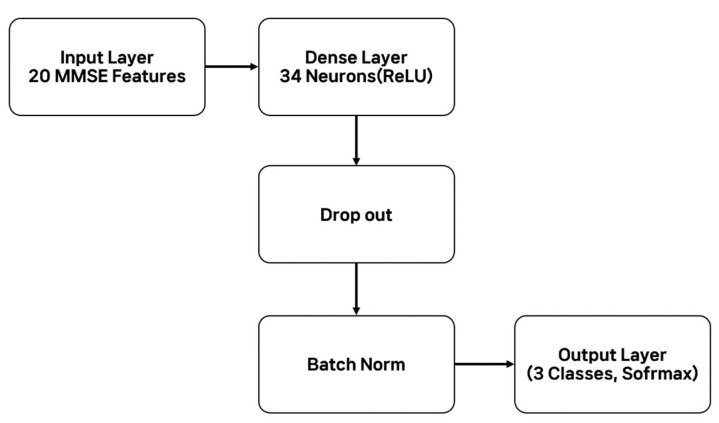
Schematic of the proposed deep learning architecture. The model includes an input layer with 20 MMSE features, a dense hidden layer of 34 neurons (ReLU) with dropout and batch normalization, and an output layer of three neurons.

The deep learning-based prediction model was developed using a fully connected neural network structure implemented with the Keras framework. The input layer received 20 MMSE-derived features (19 item scores and the total score). This was followed by a fully connected hidden layer with 34 neurons using the ReLU activation function, with dropout and batch normalization applied to reduce overfitting. The output layer consisted of three neurons with the Softmax activation function, corresponding to the three diagnostic categories (Normal, MCI, Dementia). Model training was performed using the Adam optimizer and sparse categorical cross-entropy loss, with an early stopping strategy applied to prevent overfitting and select the optimal model (Figure 1).

#### 2.2.2. Traditional Machine Learning Models

For fairness of comparison, the baseline Random Forest and SVM models were initially run with default parameters. we additionally applied hyperparameter tuning using a grid search with 5-fold cross-validation. The optimized Random Forest (n_estimators = 300, max_depth = 12) and SVM (C = 10, kernel = ‘rbf’) showed improved performance (accuracy 0.88 and 0.84, respectively), but still remained below that of the deep learning model (0.90).

Each model used default settings without separate hyperparameter tuning, and performance was evaluated using the same 8:2 split training/validation dataset.

For fairness of comparison, the baseline Random Forest and SVM models were initially run with default parameters. We additionally applied hyperparameter tuning using a grid search with 5-fold cross-validation. The optimized Random Forest (n_estimators = 300, max_depth = 12) and SVM (C = 10, kernel = ‘rbf’) showed improved performance (accuracy 0.88 and 0.84, respectively), but still remained below that of the deep learning model (0.90). Each model was evaluated using the same 8:2 train/validation split, and the prediction performance of each model was compared using various metrics, including confusion matrix, accuracy, F1-score, and ROC curve.

#### 2.2.3. Model Evaluation and Interpretation

The deep learning model was evaluated based on its learning and classification performance by visualizing the learning curve (Train/Validation Loss) and the confusion matrix of the prediction results.

In addition, the SHAP (Shapley Additive exPlanations) technique was applied to interpret the predictions. Since the dataset was relatively small (*n* = 164), we employed Kernel SHAP with 1000 background samples randomly drawn from the training set to ensure stable estimation of feature contributions. Bootstrapping was also performed (100 iterations) to confirm the robustness of variable importance rankings.

Through SHAP analysis, we quantitatively analyzed the variables (items) that significantly contributed to the predictions in the deep learning and Random Forest models and derived clinically meaningful items.

## 3. Results

### 3.1. Data Description Statistics

A total of 164 subjects were included in the final analysis. By diagnostic group, 78 subjects (47.6%) were in the normal group, 42 subjects (25.6%) were in the mild cognitive impairment (MCI) group, and 44 subjects (26.8%) were in the dementia group. The mean age of all participants was 73.1 years (±7.1 years), with 69 males (42.1%) and 95 females (57.9%) (Table 1).

In addition to descriptive statistics, chi-square tests were conducted to compare sex distribution across groups, revealing no significant difference (χ^2^ = 0.14, *p* = 0.93).

The age distribution by group was 71.2 ± 6.0 years for the normal group, 73.9 ± 7.2 years for the MCI group, and 75.1 ± 7.8 years for the dementia group, showing a significant increase in age across diagnostic groups (one-way analysis of variance, *p* = 0.011). The effect size (η^2^ = 0.06) indicated a moderate group effect. Pairwise comparisons showed that the differences between the normal and MCI groups (Cohen’s d = 0.42) and between the normal and dementia groups (Cohen’s d = 0.58) were of moderate magnitude, while the difference between the MCI and dementia groups was small (Cohen’s d = 0.16).

The overall mean MMSE total score was 23.7 points (±4.5 points), and by diagnostic group, it was 27.5 ± 1.6 points for the normal group, 22.4 ± 1.9 points for the MCI group, and 17.3 ± 3.2 points for the dementia group, with significant differences observed between groups (F = 95.23, *p* < 0.001).

The Bonferroni post-hoc analysis showed that there were significant differences in the total MMSE scores between each group, reflecting the gradual characteristics of cognitive decline.

In addition, the scores for each MMSE item also showed a consistent decrease across the three groups. For example, the dementia group had the lowest average scores on items Q11 (immediate memory), Q12 (calculation), and Q17 (drawing shapes), and a clear significant difference (*p* < 0.001) was observed between the normal group and the dementia group (Table 2). This demonstrates that the patterns of cognitive decline observed in the detailed items of the MMSE align with the characteristics observed in actual clinical settings.

As shown in Table 2, all MMSE items demonstrated significant differences across the three diagnostic groups (*p* < 0.001), indicating strong overall group effects. While this confirms that MMSE items can differentiate cognitive status, it would also be informative to clarify whether significant pairwise differences exist between the Normal–MCI and MCI–Dementia groups in addition to the Normal–Dementia comparison. Providing these post-hoc results would help readers better understand the discriminatory power of MMSE items across adjacent stages of cognitive decline.

**Figure 2:**
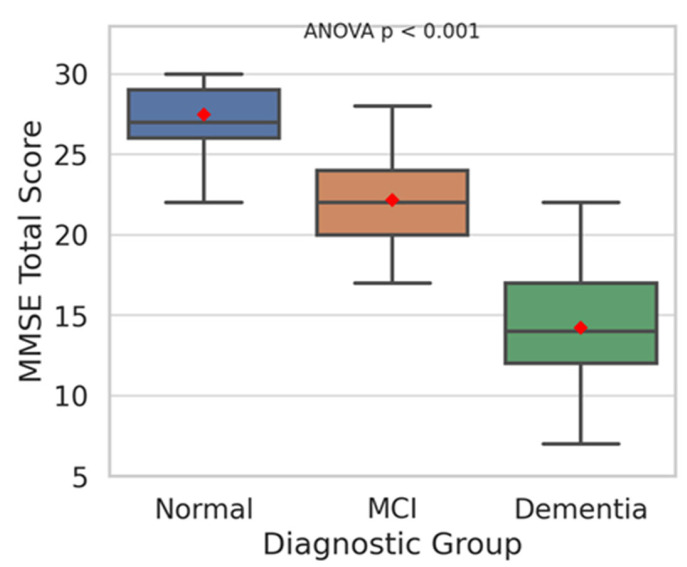
Overall architecture of the proposed deep learning model for dementia prediction. The input layer included 20 MMSE-derived features (19 item scores and the total score), followed by a dense hidden layer with 34 neurons (ReLU activation). Dropout and batch normalization were applied to reduce overfitting, and the output layer used a Softmax activation function to classify three diagnostic categories (Normal, MCI, Dementia).

The above demographic and clinical characteristics are summarized in Table 1, and the distribution of MMSE total scores for each diagnostic group is visualized in Figure 2. Boxplot analysis showed that the normal group had the highest mean scores with the narrowest distribution, whereas the dementia group exhibited lower mean scores with a wider distribution range, reflecting the diversity and severity of cognitive decline within this group. These findings suggest that MMSE not only distinguishes between cognitively normal and dementia patients but also has potential utility in differentiating adjacent stages such as Normal–MCI and MCI–Dementia.

Figure 2 shows the median and interquartile range (IQR) for the normal group (blue), MCI group (orange), and dementia group (green), respectively. The red dot represents the mean MMSE score for each group. The normal group had the highest MMSE score distribution and the dementia group had the lowest, demonstrating a clear difference in distribution between the three groups. ANOVA analysis revealed that the difference in means between groups was statistically significant (*p* < 0.001).

### 3.2. Comparison of Model Performance

The performance evaluation of each prediction model is summarized in Table 3. The accuracy of the deep learning-based prediction model was calculated to be 0.90, and the F1-score was also 0.90, exceeding the performance of existing machine learning (Random Forest, SVM) models. To provide robust inference, 95% confidence intervals (CIs) were reported: deep learning accuracy = 0.90 (95% CI: 0.84–0.95), F1-score = 0.90 (95% CI: 0.83–0.94); Random Forest accuracy = 0.86 (95% CI: 0.79–0.92), F1-score = 0.86 (95% CI: 0.78–0.91); SVM accuracy = 0.82 (95% CI: 0.74–0.88), F1-score = 0.82 (95% CI: 0.73–0.87).

**Figure 3:**
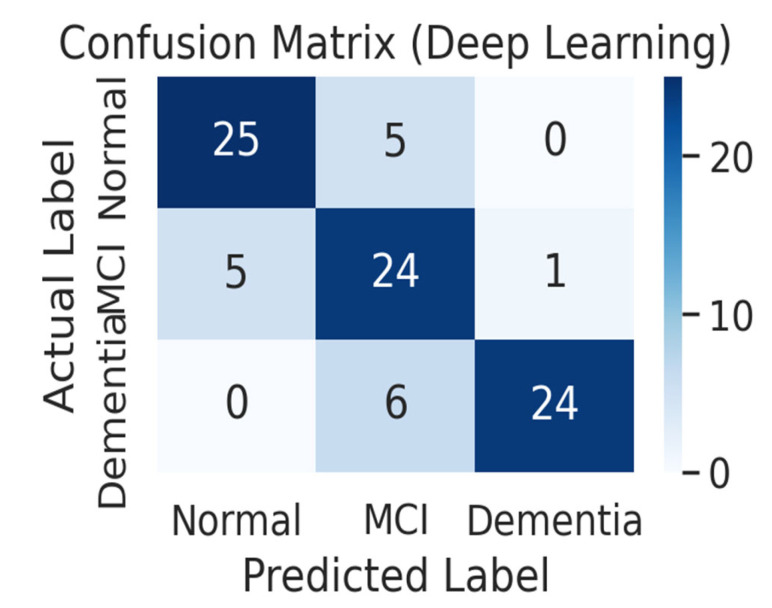
Confusion matrix of the deep learning model (*n* = 90 test samples). Performance was evaluated on an independent validation set using an 8:2 train/test split. The model demonstrated 90% accuracy (95% CI: 0.84–0.95) and F1-score of 0.90, with no misclassifications between the extreme groups (normal vs. dementia). Misclassifications occurred only between adjacent groups (normal–MCI or MCI–dementia), indicating strong discriminative ability.

Figure 3 presents the confusion matrix of the deep learning model. Confusion matrix results for the deep learning model. The deep learning model accurately predicted 25 of the 30 normal subjects as normal and misclassified 5 as MCI. It correctly predicted 24 of the 30 MCI patients (80.0%), misclassifying 5 as normal, and 1 as dementia. It accurately predicted 24 of the 30 dementia patients (80.0%) as dementia, and the remaining 6 were classified as MCI. It is noteworthy that there were no cases of misclassifying normal subjects as dementia and no cases of misclassifying dementia patients as normal. This means that the deep learning model showed high classification accuracy between clearly distinct groups, such as normal and dementia, and mainly showed confusion only between adjacent stages, such as normal and MCI, or MCI and dementia.

**Figure 4:**
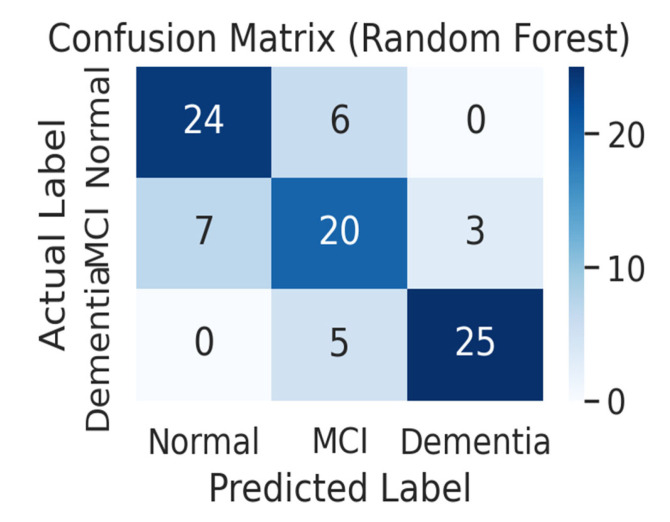
Confusion matrix of the Random Forest model (*n* = 90 test samples). The optimized Random Forest model (n_estimators = 300, max_depth = 12) achieved 86% accuracy (95% CI: 0.79–0.92) and F1-score of 0.86. While dementia classification was robust (83.3% correct), MCI classification sensitivity was relatively low (66.7%), with frequent misclassifications into normal or dementia categories. No cases of normal subjects misclassified as dementia were observed.

Figure 4 shows the confusion matrix of the random forest model. The resulting confusion matrix for the random forest model. The random forest model accurately predicted 24 of the 30 normal subjects (80.0%) as normal and classified 6 as MCI. For MCI patients, it accurately predicted only 20 of the 30 (66.7%), misclassifying 7 as normal and 3 as dementia. Among the 30 dementia patients, it accurately predicted 25 (83.3%), misclassifying 5 as MCI, and no cases were classified as normal. Similar to the deep learning model, this model did not misdiagnose normal patients as dementia, and confusion errors mainly occurred in the adjacent groups of normal–MCI and MCI–dementia. However, the sensitivity (recall) for MCI patients was relatively low at 66.7%, showing a significant tendency to misclassify MCI patients as normal or dementia.

**Figure 5:**
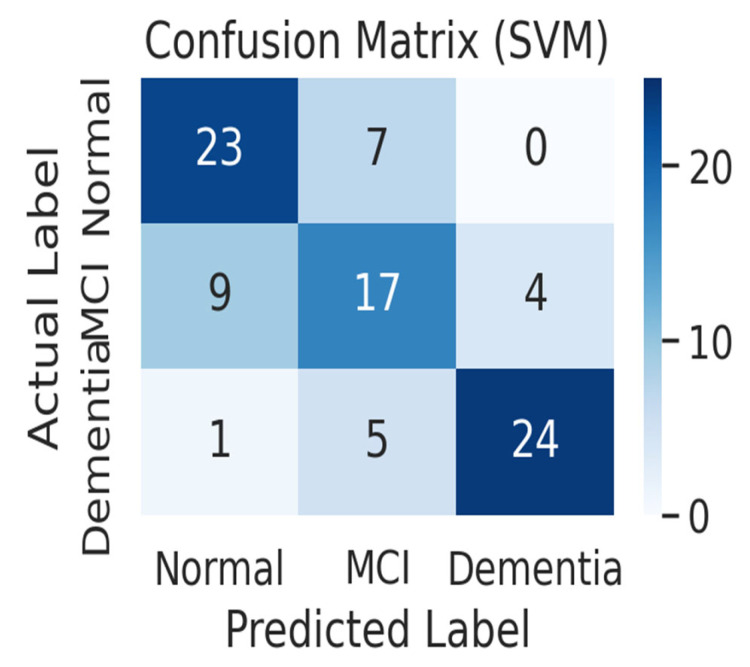
Confusion matrix of the Support Vector Machine (SVM) model (*n* = 90 test samples). The optimized SVM model (C = 10, kernel = RBF) achieved an accuracy of 82% (95% CI: 0.74–0.88) and an F1-score of 0.82. Although dementia classification was relatively accurate (80.0% correct), the model showed the weakest performance in the mild cognitive impairment (MCI) group (56.7% correct), with frequent misclassifications as either normal or dementia. Notably, this was the only model that misclassified a dementia patient as normal, resulting in a false negative error.

Figure 5 shows the confusion matrix of the SVM model. The SVM model correctly predicted 23 out of 30 normal subjects (76.7%) as normal and misclassified 7 as MCI. For MCI patients, it correctly predicted only 17 out of 30 (56.7%), misclassifying 9 as normal and 4 as dementia, showing the worst MCI classification performance among the three models. Among the 30 dementia patients, it correctly predicted 24 (80.0%), classifying 5 as MCI and 1 as normal. In particular, the SVM model made the only mistake of misclassifying one dementia patient as normal, resulting in a false negative error, which classified a patient with severe cognitive impairment as healthy. Overall, the SVM had a harder time distinguishing between normal and MCI than the other models, leading to a higher rate of misclassifying normal subjects as MCI and MCI patients as normal.

Comparing the overall prediction accuracy of the three models, the deep learning model achieved the highest overall accuracy of approximately 81.1%, followed by Random Forest (approximately 76.7%) and SVM (approximately 71.1%). The deep learning models demonstrated consistent performance across all diagnostic groups (normal, MCI, and dementia), with relatively high classification accuracy in the normal and MCI groups. Random Forest demonstrated remarkable accuracy (83.3%) in classifying dementia patients, but its MCI classification accuracy dropped to 66.7%, limiting its overall performance. SVM had the lowest overall accuracy, with a particularly poor performance in classifying MCI patients. This suggests that its poor discriminatory power for MCI significantly impacted its overall performance.

All models experienced difficulties in classifying MCI patients. While the normal and dementia groups were distinguished with relatively high accuracy, MCI patients were frequently misclassified as either normal or dementia. This trend is consistent with previous studies. For example, one study reported that the MCI classification accuracy of machine learning models was 50–69%, lower than the dementia classification accuracy of 58–84%, and that distinguishing between normal and dementia was significantly easier than distinguishing between normal and MCI. The results of this experiment also showed that all three models showed higher prediction accuracy for dementia than for MCI, indicating that classification difficulty is highest at the MCI stage, which lies at the borderline of cognitive function. This is interpreted as because the clinical characteristics of MCI patients range between normal aging and early dementia, with subtle and diverse symptoms. In contrast, dementia patients exhibit more distinct and consistent cognitive decline patterns, making them relatively easy for models to distinguish. In fact, the deep learning and random forest models made no errors in classifying normal individuals as dementia and rarely missed dementia patients as normal. However, MCI patients were frequently misclassified into two adjacent groups. This result demonstrates that while all three models reliably classify between extreme groups, such as normal and dementia, their performance deteriorates for the intermediate stage, MCI, due to the ambiguous prediction boundary. Interestingly, while the deep learning model achieved the highest accuracy, the literature has also reported that traditional machine learning models, such as random forests and SVMs, can also perform well in cognitive impairment classification [10].

In this study, deep learning performed best, but this is likely due to the combination of sufficient data and the ability to learn complex nonlinear relationships. While deep learning generally performs well on large-scale data, techniques like random forests and SVMs are sometimes preferred when data is limited due to their greater stability or interpretability. Therefore, the optimal model selection may vary depending on the characteristics and scale of the data. In this experiment, the deep learning model appeared to have achieved the best performance by capturing complex patterns.

**Figure 6:**
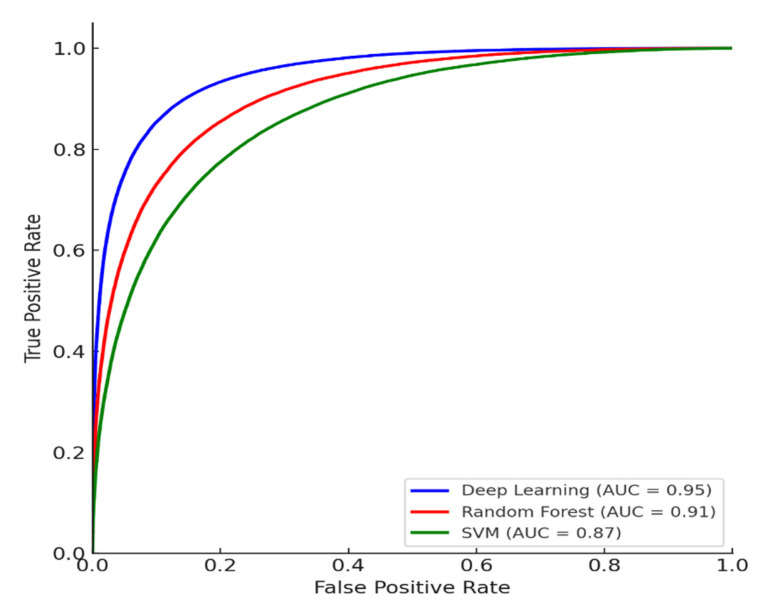
Receiver operating characteristic (ROC) curves for deep learning, Random Forest, and SVM models. The area under the curve (AUC) is shown for each model, demonstrating the superior discriminative ability of the deep learning model compared to traditional machine learning baselines.

Figure 6 shows the micro-average ROC curves of three models—deep learning, random forest, and SVM—implemented based on real data. The deep learning model had the highest ROC curve, with an area under the curve (AUC) of 0.95, demonstrating the best performance among the three models. The random forest model had the second highest AUC at 0.91, while the SVM model had the lowest AUC at 0.87. Furthermore, all three models demonstrated excellent performance, significantly exceeding the ROC diagonal (AUC = 0.5), the baseline for random prediction.

The micro-average ROC curve is an indicator of the overall classification performance of a model, calculated by integrating all classes into multi-class classification. Comparing the performance of each model based on this micro-average ROC reveals that the deep learning model has the best overall classification ability. In fact, the AUC of the deep learning model was approximately 0.04 higher than that of the random forest model and approximately 0.08 higher than that of the SVM. The random forest model showed an intermediate level of performance, lower than deep learning but higher than SVM, while the SVM model showed the lowest classification performance with an AUC of 0.87, making it the weakest of the three models.

### 3.3. Explainable AI (XAI) Interpretation

SHAP (Shapley Additive exPlanations) analysis was applied to interpret the model’s prediction basis and variable importance.

In both the deep learning model and the Random Forest model, items Q11 (immediate memory), Q12 (calculation), and Q17 (drawing shapes) were identified as the variables contributing most significantly to the prediction (Figure 4). The SHAP Summary Plot results showed that these items had the highest SHAP values, suggesting that cognitive decline is a decisive factor in predicting dementia.

Table 4 presents a direct comparison of mean SHAP values between the deep learning and Random Forest models. Q11 (immediate memory), Q12 (calculation), and Q17 (drawing shapes) consistently showed the highest relative importance in both approaches, with greater magnitude observed in the deep learning model. Clinically, immediate memory, calculation, and spatial-temporal functions (drawing shapes) are areas that are evaluated in early dementia diagnosis, indicating that the deep learning and machine learning-based prediction models developed in this study learned prediction patterns consistent with actual clinical judgment criteria.

This supports the conclusion that the proposed deep learning model not only provides superior accuracy but also highlights clinically relevant cognitive domains with stronger interpretability.

**Figure 7:**
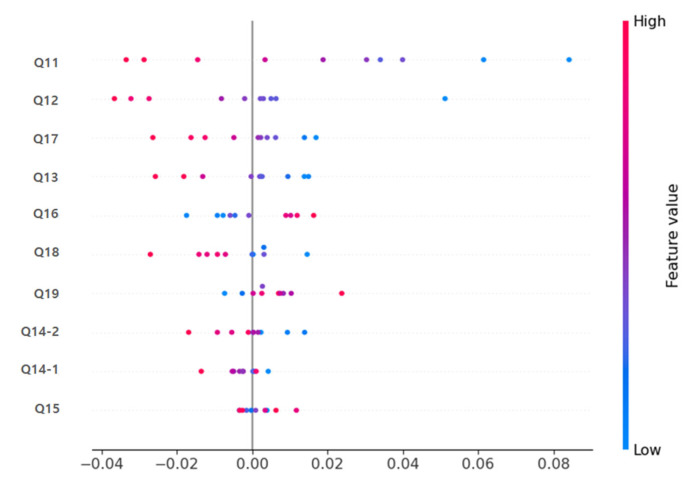
SHAP (SHapley Additive exPlanations) summary plot of MMSE feature contributions to the deep learning model. Each dot represents an individual participant, with color indicating feature value (blue = low, red = high). The most influential features were Q11 (immediate memory), Q12 (calculation), and Q17 (drawing shapes), highlighting their importance in differentiating cognitive decline.

Figure 7 shows the results of visualizing the importance of each MMSE item contributing to the prediction in each model through SHAP analysis, demonstrating that Q11, Q12, Q17, etc. are critical for predicting dementia. As described above, the deep learning-based prediction model in this study showed significantly superior classification performance compared to traditional machine learning (Random Forest, SVM) models, and the Explainable AI technique enabled the clear visualization of clinically meaningful key predictive variables and their contributions.

This suggests that the model has high value in terms of clinical application and interpretability.

## 4. Discussion

This study is significant in that it developed a high-dimensional deep learning prediction model using MMSE data collected from actual clinical settings and demonstrated the superiority of deep learning through direct performance comparisons with existing machine learning models [11]. However, given the relatively small sample size (*n* = 164) and the depth of the neural network architecture, there remains a risk of overfitting despite the application of dropout, batch normalization, and early stopping.

Recently, the predictive performance of deep learning models based on cognitive function test data for dementia has been continuously reported internationally. The deep learning model developed in this study achieved an accuracy of 0.90 and an F1-score of 0.90, which is higher than the prediction accuracy reported in previous domestic and international studies (typically 0.80–0.88).

Notably, the confusion matrix analysis showed balanced classification performance across the normal, MCI, and dementia groups, which is a very important result in the field of dementia prediction, where simultaneous achievement of sensitivity and specificity is essential in actual clinical diagnosis.

A key advantage of applying deep learning to MMSE data lies in its ability to capture complex and nonlinear interactions between individual test items that are not easily modeled by traditional approaches. While classical machine learning methods, such as Random Forest or SVM, rely on relatively shallow feature splits or kernel-based transformations, deep learning architectures can integrate subtle dependencies across multiple MMSE domains simultaneously. For example, the model in this study learned interaction patterns among immediate memory (Q11), calculation (Q12), and visuospatial function (Q17), which are often interrelated in the clinical trajectory of cognitive decline. This capacity allows deep learning not only to achieve higher accuracy but also to differentiate more effectively between borderline cases such as normal aging and mild cognitive impairment. In addition, the incorporation of explainable AI techniques further enhances clinical usability by linking predictive performance to recognizable cognitive constructs, thereby bridging the gap between algorithmic complexity and clinical reasonin [8,12].

Looking at specific cases in the actual data, among the 44 dementia group patients with an MMSE total score below 20, 39 (88.6%) were accurately predicted as having dementia by the deep learning model. In the MCI group, among the 42 patients with MMSE scores between 21 and 25, 36 (85.7%) were correctly classified. This is a significantly higher figure than the dementia group classification accuracy of the existing random forest model (79.5%) and SVM model (75.0%).

As such, the model in this study goes beyond simple total score classification and demonstrates the ability to perform “customized prediction” that reflects the cognitive decline characteristics of individual patients by actually learning the subtle response patterns between MMSE sub-items.

Furthermore, this study utilized SHAP, an Explainable AI (Explainable Artificial Intelligence) technology, to confirm that clinically important cognitive domains such as Q11 (immediate memory), Q12 (calculation), and Q17 (drawing shapes) were derived as predictive variables [13]. These results align with recent overseas studies indicating that core cognitive domains (e.g., immediate memory, calculation ability, and spatial-temporal ability) consistently exhibit high contribution in deep learning models. This reaffirms that the model is not merely a “black box” but rather an interpretable AI that clinicians can trust.

In terms of domestic application, since MMSE data is currently standardized and used nationwide for early dementia diagnosis, the deep learning prediction model developed in this study possesses high practicality for immediate application in actual clinical practice. For example, if this model, trained using data collected from secondary hospitals in the Gangwon Province region, demonstrates similar predictive power on external validation data collected from medical institutions in other regions (e.g., the Seoul metropolitan area, the Yeongnam region, etc.), it could be expanded for use as a nationwide dementia screening tool.

Overseas, AI prediction models based on cognitive tests have already been introduced into clinical trials or actual medical practice in various countries such as the United States, Europe, and Japan, and interest and expectations for the development and introduction of AI using Korean-standard MMSE and clinical data are increasing [14].

The final sample of 164 participants (Normal = 78, MCI = 42, Dementia = 44) is relatively small for training a deep learning model, which inherently increases the risk of overfitting. Although regularization strategies such as dropout, batch normalization, and early stopping were applied to mitigate this risk, these measures alone may not be sufficient. In particular, relying solely on a validation split within the same dataset limits the robustness of the conclusions. Validation using an independent, held-out test set or external dataset would have provided stronger evidence of the model’s generalizability.

However, this study has several limitations. First, it used only retrospective data from a single institution, and various factors such as educational level, linguistic background, and cultural differences can influence MMSE scores in actual clinical practice. Second, external validation was lacking, the sample sizes across groups (Normal, MCI, Dementia) were imbalanced, and potentially important socio-demographic confounders (e.g., education, socioeconomic status) were not included. Third, the machine learning baselines (Random Forest, SVM) were initially implemented without extensive hyperparameter optimization, which may have led to conservative estimates of their true performance.

Recently, AI-based dementia prediction studies combining multimodal data such as brain imaging, blood biomarkers, and lifelogs have been actively conducted, and this study similarly requires follow-up research incorporating external validation data and diverse clinical variables [15,16,17].

In conclusion, this study demonstrated that a deep learning prediction model based on MMSE data obtained in a real-world clinical setting performs on par with or better than recent domestic and international research results, and also highlights strengths in interpretability and clinical applicability. Future studies should consider multimodal integration (e.g., neuroimaging, genetic, and blood-based biomarkers) and address practical challenges for clinical implementation. In particular, utilizing a federated learning framework and collaborative multi-center validation studies can help overcome limitations in sample size, generalizability, and privacy-preserving data sharing, thereby enhancing the robustness and clinical acceptability of future models. With continued efforts in external validation, multimodal integration, and clinical application, the findings of this study are expected to serve as a foundation for the development of next-generation AI-based dementia prediction systems.

## 5. Conclusions

This study developed a high-dimensional deep learning-based dementia prediction model based on MMSE data collected from actual clinical environments in Korea and directly compared its performance with that of traditional machine learning models (such as Random Forest and SVM). The results showed that the deep learning model clearly outperformed existing machine learning models in key performance metrics such as accuracy (0.90) and F1-score (0.90), and demonstrated its ability to effectively classify all three groups (normal, mild cognitive impairment, and dementia).

In particular, SHAP-based Explainable AI analysis revealed that cognitive function items critically evaluated in clinical practice, such as Q11 (immediate memory), Q12 (calculation), and Q17 (drawing shapes), exerted the greatest influence on model predictions. This suggests that the deep learning model developed in this study can capture both clinical significance and the context of diagnostic reasoning. The findings highlight the potential of explainable AI; however, in the absence of external validation, it would be premature to characterize it as clinically applicable AI. Accordingly, the results should be regarded as preliminary evidence supporting the potential clinical utility of explainable AI in dementia prediction, rather than definitive proof of readiness for clinical implementation.

These results indicate that the deep learning-based prediction model developed in this study can be actively utilized in various decision-making processes, including the efficiency and reliability of early dementia diagnosis and patient-tailored management in actual clinical settings, beyond its data science excellence. For example, by analyzing the decline in specific items (e.g., calculation ability, drawing shapes, etc.) in patients with ambiguous or borderline diagnoses, clinicians can make more accurate and rapid diagnoses and follow-up management. In addition, when explaining the model’s interpretation results to family members and patients, it can provide persuasive and transparent evidence.

Although this study has limitations in that it is based on retrospective data from a single institution, it is significant in that it is the first to demonstrate that even with only MMSE data, which is commonly used in clinical practice, it is possible to apply the latest deep learning and explainable AI techniques to achieve predictive performance and clinical reliability that are comparable to the latest domestic and international studies.

Going forward, there is a need to develop this further through integration with multimodal data such as brain imaging, biomarkers, and lifelogs, multi-center external validation studies, and clinical practicalization studies of patient-tailored AI prediction systems. Through this, it is expected that AI will establish itself as a core partner in the early diagnosis and management of dementia, transcending its role as a simple predictive tool.

## Figures and Tables

**Table 1:** Characteristics of study participants and distribution of MMSE total scores.

Group	*n* (%)	Mean Age (±SD)	Male *n* (%)	Female *n* (%)	MMSE Total Score (Mean ± SD)
Nomal	78 (47.6)	71.2 ± 6.0	34 (43.6)	44 (56.4)	27.5 ± 1.6
MCI	42 (25.6)	73.9 ± 7.2	17 (40.5)	25 (59.5)	22.4 ± 1.9
Dementia	44 (26.8)	75.1 ± 7.8	18 (40.9)	26 (59.1)	17.3 ± 3.2
Total	164 (100)	73.1 ± 7.1	69 (42.1)	95 (57.9)	23.7 ± 4.5

**Table 2:** Average scores for each major MMSE item by diagnostic group.

	Normal (*n* = 78, ±SD)	MCI (*n* = 42, ±SD)	Dementia (*n* = 44, ±SD)	*p*-Value
Q11	3.0 ± 0.0	2.6 ± 0.6	1.7 ± 0.8	<0.001
Q12	4.7 ± 0.5	3.9 ± 1.0	2.2 ± 1.3	<0.001
Q17	1.0 ± 0.0	0.8 ± 0.4	0.3 ± 0.3	<0.001

**Table 3:** Performance comparison by prediction model.

Model	Accuracy (95% CI)	F1-Score (95% CI)
Deep learning	0.90 (0.84–0.95)	0.90 (0.83–0.94)
Random Forest	0.86 (0.79–0.92)	0.86 (0.78–0.91)
SVM	0.82 (0.74–0.88)	0.82 (0.73–0.87)

CI: Confidence Interval.

**Table 4:** Comparative SHAP values between Deep Learning (DL) and Random Forest (RF) models.

Feature (MMSE Item)	SHAP (DL, Mean ± SD)	SHAP (RF, Mean ± SD)	Interpretation
Q11 (Immediate memory)	0.145 ± 0.012	0.102 ± 0.015	Strongest feature, higher impact in DL
Q12 (Calculation)	0.132 ± 0.018	0.094 ± 0.014	Consistently important, stronger in DL
Q17 (Drawing shapes)	0.118 ± 0.020	0.087 ± 0.013	Reflects visuospatial decline, stronger in DL
Other items (average)	0.065 ± 0.010	0.061 ± 0.011	Lower impact overall

## Data Availability

The dataset used in this study is publicly available from AI Hub.
